# Overexpression of Peony *PoWOX1* Promotes Callus Induction and Root Development in *Arabidopsis thaliana*

**DOI:** 10.3390/plants14121857

**Published:** 2025-06-17

**Authors:** Xue Zhang, Tao Hu, Yanting Chang, Mengsi Xia, Yanjun Ma, Yayun Deng, Zehui Jiang, Wenbo Zhang

**Affiliations:** 1 International Center for Bamboo and Rattan, No. 8, Futong Eastern Avenue, Wangjing Area, Chaoyang District, Beijing 100102, China; zx949949@126.com (X.Z.); hutao@icbr.ac.cn (T.H.); changyanting@icbr.ac.cn (Y.C.); mayanjun@icbr.ac.cn (Y.M.); yayundeng@icbr.ac.cn (Y.D.); jiangzh@icbr.ac.cn (Z.J.); 2 Key Laboratory of National Forestry and Grassland Administration/Beijing for Bamboo & Rattan Science and Technology, No. 8, Futong Eastern Avenue, Wangjing Area, Chaoyang District, Beijing 100102, China; 3 Pingxiang Bamboo Forest Ecosystem Research Station, Pingxiang 532600, China; 4 Sichuan Academy of Forestry Sciences, Chengdu 610081, China; xiamengsi@icbr.ac.cn

**Keywords:** *WOX1*, peony, root development, callus

## Abstract

Plant-specific WUSCHEL (WUS)-related homeobox (WOX) family of transcription factors are involved in apical meristem maintenance, embryogenesis, lateral organ development, and hormone signaling. Among the members of this family, WOX1 is known to play essential roles in many species. However, the function of the peony ‘Feng Dan’ (*Paeonia ostii* L.) *WOX1* (*PoWOX1*) remains unknown. The initial bioinformatic analysis revealed that *PoWOX1* belongs to the modern clade of the *WOX* gene family and has a highly conserved homeodomain (HD), the WUS motif, the STF-box, and the MAEWEST/WOX4-box. Subsequent heterologous overexpression in *Arabidopsis thaliana* revealed that *PoWOX1* promotes root growth, early shoot initiation, and flowering. The root vascular tissues, especially the arrangement and size of xylem cells, were different between the *PoWOX1*-overexpressing transgenics and the wild-type plants, and the pericycle cells adjacent to the xylem divided more easily in the transgenics than in the wild type. Furthermore, under in vitro conditions, the transgenic leaf explants exhibited more callus induction and differentiation than the wild-type leaf explants. Thus, the study’s findings provide novel insights into the role of *PoWOX1* in promoting root development and callus tissue induction and differentiation, serving as a reference for developing an efficient regeneration system for the peony.

## 1. Introduction

Peony ‘Feng Dan’ (*Paeonia ostii* L.), belonging to the Paeoniaceae family, *Paeonia* genus, and Paeonia group (Section Moutan DC), is a deciduous subshrub species native to China. It is recognized as one of the top ten traditional flowers in China due to its large and vibrant blooms. It is also well-known for its medicinal and oil-producing properties [1,2,3]. Selective breeding and crossbreeding are the common methods used to propagate and improve peonies. However, the long growth period of peonies has limited the progress and use of these techniques in the propagation of this species. Under such a scenario, tissue culture is the preferred method of propagation. The high micropropagation rate and rapid generation of propagules help retain the mother plant’s excellent traits. Currently, tissue culture is used in several species for large-scale plant propagation, cultivar selection, and genetic breeding [2,4].

Regeneration in tissue culture occurs via somatic embryogenesis or de novo organogenesis [5]. During somatic embryogenesis, explants are induced to form calluses, which further differentiate into embryos and form new plants. On the other hand, during de novo organogenesis, the cells of explants directly form new plant parts. Thus, these two pathways primarily differ in terms of whether or not the somatic cells are induced to form embryos [6]. Establishing an efficient regeneration system is the most crucial step in tissue culture [4]. However, in peonies, recalcitrance has led to low shoot differentiation, rooting difficulties, and vitrification complications, hindering success in tissue culture [7].

Research has demonstrated that the ectopic expression of essential transcription factors (TFs) induces somatic embryogenesis [8,9,10,11,12]. Most importantly, the *WUSCHEL (WUS)-related homeobox* (*WOX*) genes induce somatic embryogenesis and meristem tissue formation [13]. These genes form a network alongside cell-specific marker genes, such as *Somatic Embryogenesis Receptor-like Kinase* (*SERK*) and *AGAMOUS-LIKE15* (*AGL15*), and regulate embryogenic development. *Leafy cotyledon* (*LEC*) genes and their activated family members (*LEC1*, *LEC2*, *FUS3*, *ARF*, and *ABI3*) also participate in these networks [14,15,16,17,18,19,20]. Moreover, the members of the *WOX* gene family have been proven to exhibit functional redundancy and perform specific and compensatory roles [21]. Thus, we consider WOXs as crucial factors influencing plant zygotic embryogenesis [22] and candidates for manipulating the regeneration process.

The *WOX* gene family is a superfamily of homeobox (HB) TFs found in plants. Members of this family have a conserved homeodomain (HD), which comprises 60 to 66 amino acids that fold into a helix–turn–helix structure and recognize and bind with specific DNA sequences [22,23,24,25,26,27]. In addition, the *WOX* TFs have four specific motifs: WUS-box, EAR-like motif, STF-box, and MAEWEST/WOX4-box [26,28]. The WUS-box is found near the C-terminus in the members of the WUS/modern branch and is necessary for the TFs’ repressive activity. The EAR-like motif is found at the C-terminus of WUS, WOX5, and WOX7 homologs and contributes to the inhibitory activity; however, this contribution is dispensable for WUS function. The STF-box is found at the C-terminus of the homologs of WOX1 and WOX6 and functions antagonistically to STF (STENOFOLIA) during leaf and flower development. The MAEWEST/WOX4-box, located at the N-terminus of HD, is present only in the homologs of WOX1 and WOX4, and its function remains unknown [22,26,29,30].

It is known that the members of the WOX family play significant roles in diverse plant processes, such as apical meristem maintenance, stem cell regulation, lateral and floral organ formation, embryonic development, hormone signal transduction, and stress resistance by specifically regulating cell proliferation and differentiation [27,28,31,32]. In Arabidopsis, *AtWOX1* regulates leaf development, primarily controlling the proliferation of lateral organs, while *AtWOX1* and *AtWOX3* regulate the lateral growth of leaves [33,34]. In tomatoes (*Solanum lycopersicum* L.), the WOX1 homolog gene, *SlLAM1*, promotes the expansion of various leaf types. It regulates the outward growth of leaves, especially the mid-lateral leaves, and the initiation of secondary leaves. Additionally, *SlLAM1* influences the growth of floral organs and affects the fertility of gametophytes [26,35]. In roses, *RcWOX1* is expressed during callus formation. The ectopic overexpression of *RcWOX1* in Arabidopsis significantly enhanced lateral root formation [36]. Similarly, *JsWOX1* and *JsWOX4* are expressed in the callus tissues of jasmine (*Jasminum sambac* L. Aiton). The overexpression of *JsWOX1* induced root differentiation in jasmine callus tissues [6]. These studies established that the *WOX* gene family regulates meristematic tissues, such as the shoot apical meristem (SAM), root apical meristem (RAM), and cambium layer; however, their role in the regeneration of woody plants, such as the peony, remains undetermined.

Therefore, the present study investigated the function of the peony *PoWOX1* gene. We overexpressed *PoWOX1* in *Arabidopsis thaliana* and analyzed its growth and development. We examined callus initiation, differentiation, and root cell division in explants of *PoWOX1*-overexpressing Arabidopsis.

## 2. Results

### 2.1. Bioinformatic Analysis of PoWOX1

**Figure 1:**
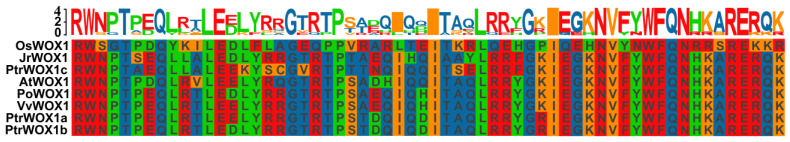
Multiple sequence alignment of the WOX1 proteins from peony and various other plant species. The highly conserved homeodomain (HD) of WOX1 proteins from six species is shown in the upper panel.

**Figure 2:**
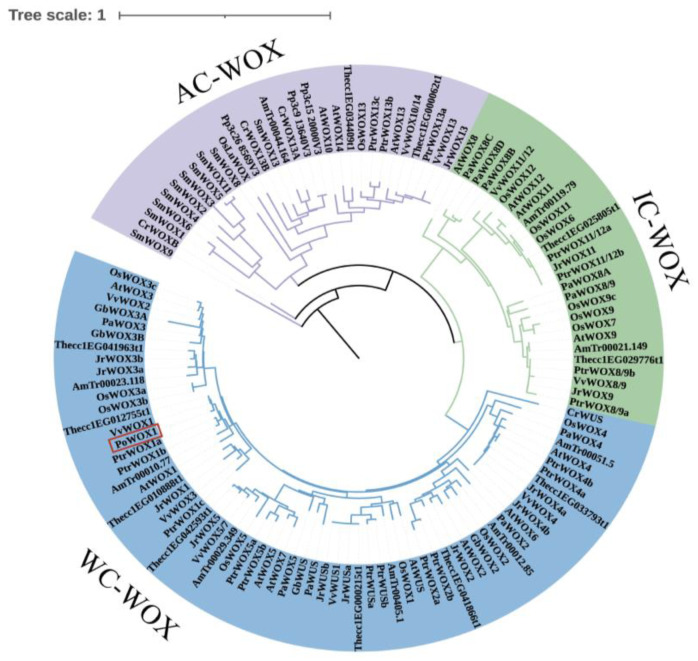
Phylogenetic analysis based on the WOX1 proteins of peony and other species. The neighbor-joining phylogenetic tree was constructed using the amino acid sequences of PoWOX1 and the WOX homologous proteins of 13 other species. PoWOX1 is shown in a red rectangular box in the tree.

**Figure 3:**
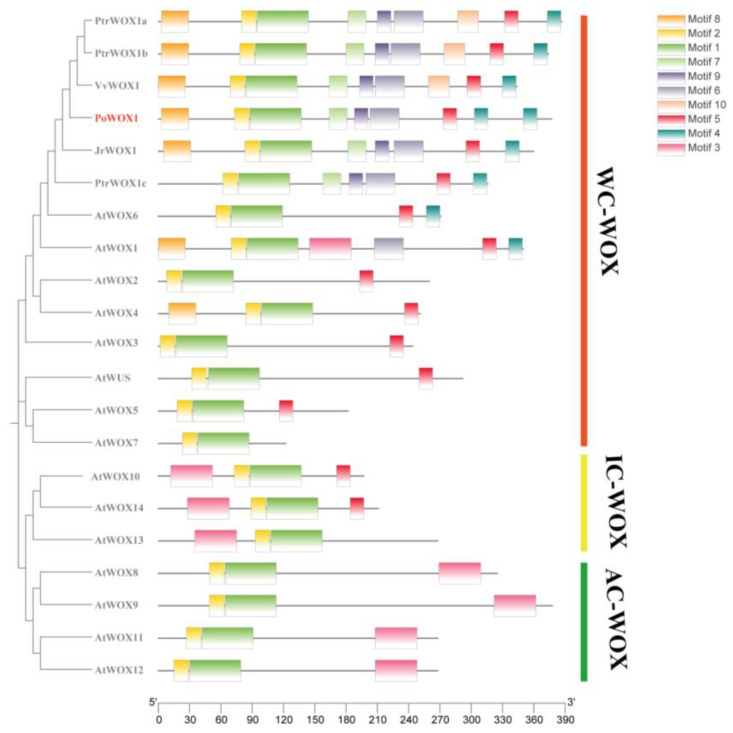
Conserved motifs in the peony PoWOX1. The motifs of PoWOX1 were identified by comparing them with the known WOX members of *Arabidopsis thaliana*, *Juglans regia*, *Vitis vinifera*, and *Populus trichocarpa.* The rectangular blocks in different colors represent different motifs within the protein sequence.

An initial comparison of the WOX1 proteins from six species showed the presence of the highly conserved HD of the WOX gene family in PoWOX1 (Figure 1). Phylogenetic analysis using the amino acid sequences further classified the WOX proteins of peony and 13 other species into three branches: a modern clade (WC-WOX), an intermediate clade (IC-WOX), and an ancient clade (AC-WOX). As per this classification, PoWOX1 was included in the modern clade and was most closely related to the WOX protein of grape (*Vitis vinifera*) (Figure 2). Subsequent analysis based on the motifs grouped the PoWOX1, PtrWOX1 (*Populus trichocarpa*), VvWOX1 (grape), JrWOX1 (*Juglans regia*), and 15 AtWOX (*Arabidopsis thaliana*) members into three branches, with PoWOX1 grouped into the modern clade. This analysis also revealed the presence of the conserved HD (motifs 1 and 2), WUS-box (motif 5), STF-box (motif 4), and MAEWEST/WOX4-box (motif 8) in PoWOX1 (Figure 3).

### 2.2. Phenotypic Analysis of Arabidopsis Plants Overexpressing PoWOX1

**Figure 4:**
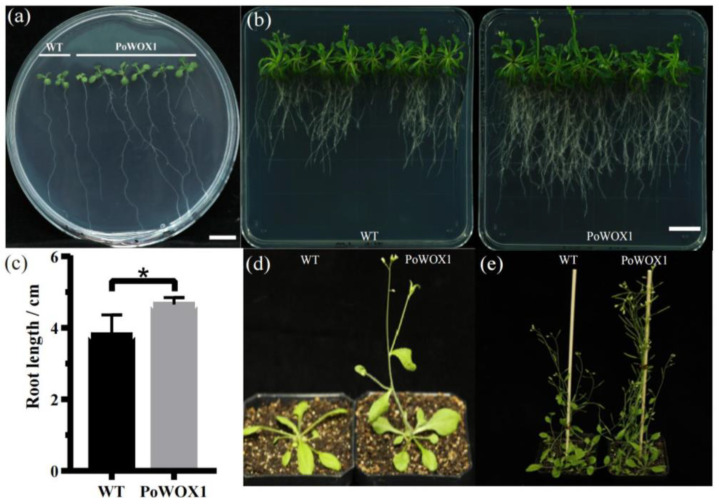
The phenotype of the wild-type and *PoWOX1*-overexpressing Arabidopsis plants. (**a**) Lateral root phenotype of Arabidopsis plants grown under aseptic conditions on day 18. (**b**) Root phenotype of Arabidopsis plants on day 32 under aseptic conditions. (**c**) Bar graph showing the maximum root length of Arabidopsis plants on day 32 under aseptic conditions. (**d**,**e**) The phenotype of Arabidopsis plants grown in soil. * stands for 0.1% level prominent. The scale bar is 5 mm.

Furthermore, *PoWOX1* was overexpressed in Arabidopsis to explore the gene’s role in plant growth and development. Under aseptic conditions, the transgenic Arabidopsis seedlings overexpressing *PoWOX1* produced significantly longer roots than the wild-type seedlings on day 7 (Figure 4a). By day 32, these lines exhibited significantly longer roots and higher lateral root density than the wild-type plants (Figure 4b,c).

Meanwhile, in the soil, the seeds of the Arabidopsis plants overexpressing *PoWOX1* produced shoots and flowers by day 25, whereas those of the wild type produced these organs only by day 29. At the flowering stage, the transgenics were significantly taller than the wild-type plants. By day 40, the transgenic lines demonstrated faster growth than the wild-type plants. Thus, the experiments proved that *PoWOX1* promoted the development of Arabidopsis from the vegetative stage to the reproductive stage; it specifically promoted plant growth, early shoot initiation, and flowering.

### 2.3. Assessment of PoWOX1 Expression in Arabidopsis Transformants

**Figure 5:**
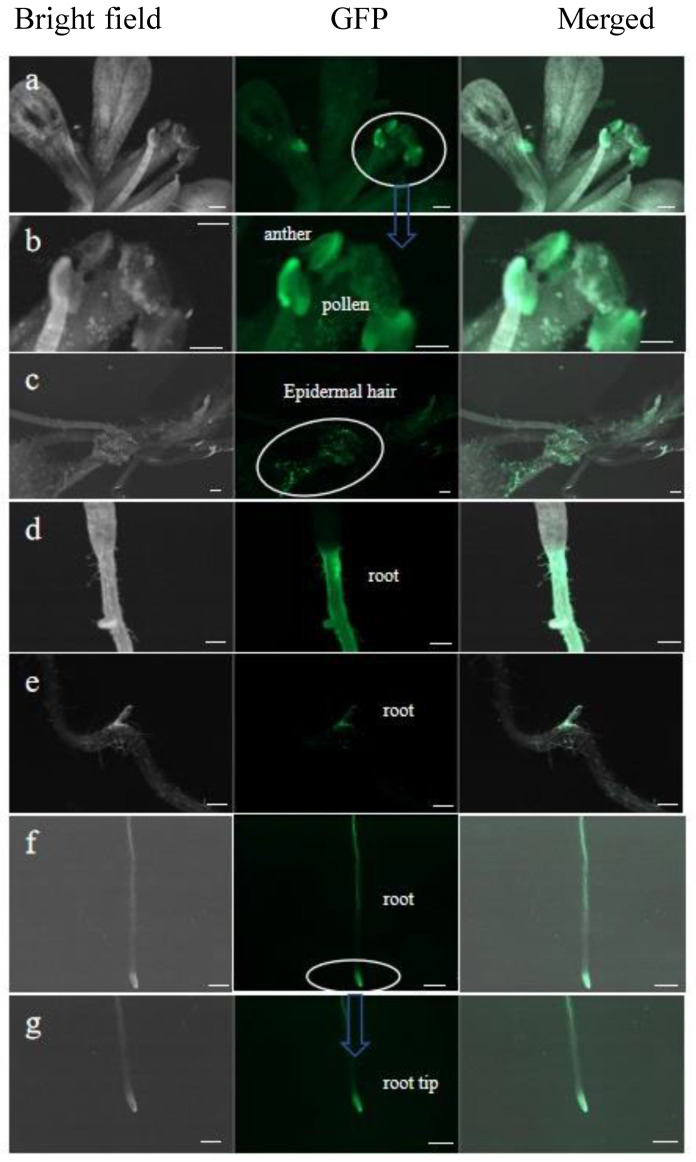
Expression of *PoWOX1* in the transgenic Arabidopsis. Images show the expression of PoWOX1 fusion protein in (**a**,**b**) anthers and pollen, (**c**) epidermal hairs, (**d**–**f**) roots, and (**g**) root tips. The bright field, fluorescence (green fluorescent protein, GFP), and merged (bright field, GFP) images are shown. The green fluorescence indicates the expression of the PoWOX1 fusion protein. The white circle and blue arrows represents the enlarged area. The scale bar shown is 200 μm.

The seeds of Arabidopsis transformants were cultured aseptically, and the fluorescence due to the PoWOX1 fusion protein was analyzed using a Zeiss Axio Zoom microscope. This analysis detected PoWOX1 expression in the anthers, pollen, and epidermal hairs of transgenic Arabidopsis (Figure 5a–c), suggesting its role in reproductive growth and epidermal hair development. PoWOX1 expression was also detected in the primary root, lateral root, and root tip (Figure 5d–g), with an intense expression in the lateral root primordium. This observation indicated the role of *PoWOX1* in regulating root growth, primary root development, and lateral root growth in Arabidopsis.

### 2.4. Analysis of Arabidopsis Roots Overexpressing PoWOX1

**Figure 6:**
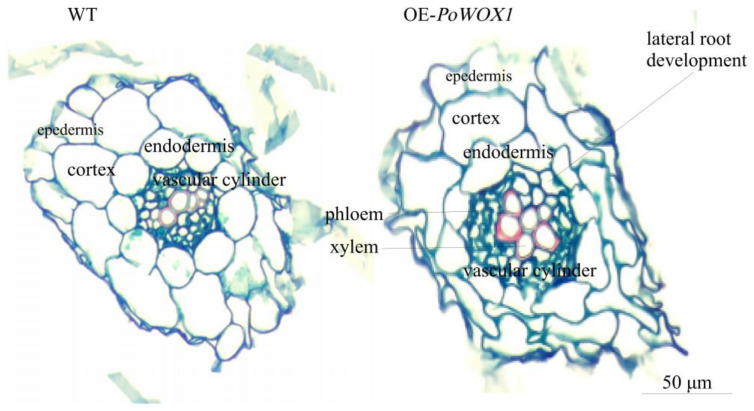
Microscopic analysis of the roots of *PoWOX1*-overexpressing and wild-type Arabidopsis plants. The cross-sections were obtained from a segment 3–5 cm above the root tip of Arabidopsis grown for about 30 days in soil. The images show the epidermis, cortex, endodermis, and vascular cylinder from outside to inside. WT indicates the wild type, while PoWO indicates the transgenic line. Cells shown in the red circle are the large and highly divisible pericycle cells near the xylem of the transgenic Arabidopsis roots. The scale bar shown is 50 μm.

Further, to investigate the cytoarchitectural differences between the roots of *PoWOX1*-overexpressing transgenic plants and the wild-type plants, cross-sections of their root tissues were analyzed. This analysis revealed no significant difference in the epidermis and endodermis of the root system between the wild-type and Arabidopsis transformants. In contrast, a significant difference was detected in the center pillar between the two. The xylem cells in the vascular cylinder were arranged in a ring pattern in the transgenic roots but in a linear pattern in the wild-type roots (Figure 6). Moreover, the xylem cells in the transgenic roots were relatively bigger than those in the wild-type roots. The pericycle cells near the xylem were larger and highly divisible (marked by red circles) in the transgenics compared to the wild-type plants (Figure 6).

### 2.5. Callus Tissue Induction and Differentiation from the Leaves and Roots of PoWOX1-Overexpressing Arabidopsis

**Figure 7:**
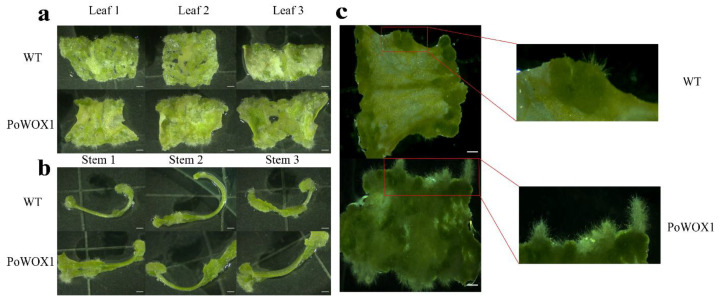
Callus induction from the leaves and stems of *PoWOX1*-overexpressing and wild-type Arabidopsis. (**a**,**b**) Healing tissue induction from the leaves and stems of wild-type and transgenic plants cultured on CIM for 7 days in the dark and SIM for 7 days in the light. (**c**) Healing tissue induction from the leaves of wild-type and transgenic plants cultured on CIM for 7 days in the dark and SIM for 17 days in the light. The scale bar is 1 mm.

Finally, to explore the function of *PoWOX1* in the regeneration process, we cultured leaf and stem pieces of wild-type and transgenic plants on a callus induction medium (CIM) in the dark for a fortnight to induce healing and then transferred them to shoot induction medium (SIM) and cultured them for a week. In the SIM, the transgenic leaf explants showed induction of more callus tissues than the wild-type leaf explants (Figure 7a). Meanwhile, the stems of wild-type and transgenic plants demonstrated similar induction of callus tissues (Figure 7b). Additionally, the callus tissues formed from the leaves of the transgenics were more prone to differentiate into roots than those of the wild-type plants (Figure 7c). These results indicated that *PoWOX1* promoted the induction of callus tissues and their differentiation into roots in Arabidopsis.

## 3. Discussion

Peonies are species with significant ornamental and medicinal values; however, the challenges associated with in vitro callus induction and root differentiation from the somatic embryos have limited their large-scale production [37,38]. Studies in various plant species have proven the role of the WOX TF family in leaf development, flower development, callus tissue induction, proliferation, and root regeneration in various species [6,22,26,39].

Bioinformatics analysis revealed that PoWOX1 contains the WOX gene family’s highly conserved HD, the WUS motif, the STF-box, and the MAEWEST/WOX4-box. Subsequent phylogenetic analysis revealed that PoWOX1 belongs to the modern clade, consistent with the WOX1 proteins of other species [6,22,40]. This classification was validated based on the WUS-box, which distinguishes members of the modern clade from those of the other branches and is necessary for the TFs’ repressive activities. Additionally, the PoWOX1 contained the STF-box, found in WOX1 and WOX6 homologs and known for its inhibitory role, and the MAEWEST/WOX4-box, present in WOX1 and WOX4 homologs [26,27,41]. These similarities suggested a doubling event consistent with their ancestors and indicated that PoWOX1 functions like the WOX proteins of other species [42,43].

Various TFs have been identified to regulate the regeneration of shoots, roots, and embryo-like structures from explants by stimulating the stem cells to establish the apical meristematic tissue primordia. In adult plants, the stem cells are distributed throughout the body along the vascular system. The primary cell populations are found within the stem apical meristem (SAM) and root apical meristem (RAM), while the mesophyll sheath cells adjacent to the xylem of roots are meristematic [44,45]. In this study, the Arabidopsis lines overexpressing *PoWOX1* grew more rapidly and produced shoots and flowers earlier than the wild-type plants, suggesting that *PoWOX1* promotes SAM development and drives growth. The overexpression of *PoWOX1* promoted root growth and development and lateral root production. This observation is consistent with the increased lateral root density observed in Arabidopsis overexpressing the rose *RcWOX1* [36]. These results indicated that PoWOX1 is important for regeneration in the peony.

Subsequent analysis showed significant differences in the arrangement and size of the xylem cells between the transgenic roots and the wild-type roots in plants obtained from the seeds. Compared with the wild type, the transgenics had relatively more divisible pericycle cells adjacent to the root xylem, which probably promoted lateral root formation from the lateral root primordia [46]. Generally, lateral roots develop from the pericycle cells; these cells first form the lateral root primordia through periplasmic division and, ultimately, the lateral roots. Thus, the data of the study suggested that PoWOX1 promotes vascular tissue morphogenesis and regulates root development and growth by manipulating the apical meristem.

Furthermore, tissue culture experiments revealed that the leaf explants of the transgenics induced more callus tissues and differentiation into roots (Figure 6), suggesting a significant role for *PoWOX1* in regulating in vitro regeneration. In jasmine (*Jasminum samba*), JsWOX1, which acts transcriptionally upstream of JsWOX4 and JsWOX13, regulates root primordia initiation. Overexpression of this gene in *Jasminum samba* led to more rooted callus tissues and roots per callus tissue [6], consistent with our observations. Studies have also indicated that the functional balance between the *WOX1* gene and phytohormones (auxin and cytokinins) is a key factor controlling cell proliferation in Arabidopsis. In an integrated manner, WOX1 and auxin act to promote cell proliferation and increase plant biomass [40]. Therefore, we speculate that PoWOX1 enhances callus differentiation probably by regulating differentiation-related genes and hormone synthesis. For instance, the rose *RcWOX1* induced the upregulation of PIN-FORMED 1 (PIN1) and PIN-FORMED 7 (PIN7) genes and promoted hormone synthesis, resulting in root differentiation of callus tissues [36]. Thus, the study’s findings indicate that PoWOX1 promotes callus induction and enhances root differentiation; however, the detailed mechanism needs to be investigated.

In the Arabidopsis transgenics, the expression of *PoWOX1* was detected in anthers, pollen, epidermal hairs, primary roots, lateral roots, and root tips. Research has proven that the *WUS* gene is important for anther growth and development in Arabidopsis [47]. Similarly, in tomato, *SlWOX1*, which is expressed in the anthers and pollen, regulates floral growth and gametophyte fertility. Thus, these earlier reports, combined with the detection of *PoWOX1* expression in the reproductive organs, suggested that it regulates the development of the floral organs in peonies. In addition, fluorescence due to PoWOX1 in the primary root, lateral root, and root tip of the transgenic Arabidopsis suggested its importance in regulating root development. Real-time PCR revealed differences in *PoWOX1* expression among the plant parts; *PoWOX1* expression was high in the leaves but low in the stems and roots. Similarly, Xia et al. detected high *PoWOX1* expression in the leaf parts of peony histocultures [48]. Thus, our observations suggested a function for *PoWOX1* in the leaves, consistent with Arabidopsis AtWOX1 and its homologous genes that regulate the proliferation of lateral organs [33,34]. Our in vitro experiments that revealed the induction of more callus tissues from the transgenic leaf explants than the wild-type leaf explants supported this hypothesis. Thus, the observations of the present study confirm that PoWOX1 regulates the proliferation of lateral organs and the differentiation of callus tissues.

## 4. Materials and Methods

### 4.1. Plant Material and Cultivation Conditions

The *Arabidopsis thaliana* Columbia (Col-0) ecotype was used in this study to generate the transgenics. The plants of this ecotype were grown in pots containing a mixture of grass charcoal and vermiculite (2:1) under the following conditions: a light/dark cycle of 16 h/8 h, a temperature of 24 ± 1 °C, and a relative humidity of 60~70%.

### 4.2. Construction of the Plant Expression Vector and Transformation of Agrobacterium Cells

The 35S::PoWOX1-EGFP plant expression vector (provided by Weidi, Shanghai, China) was introduced into the *Agrobacterium tumefaciens* strain GV3101 using the heat shock method [48]. About 0.5 μg of the plasmid was added to 100 μL of GV3101 competent cells and mixed gently by tapping the bottom of the tube. The tube was then left on ice for 5 min, frozen in liquid nitrogen for 5 min, placed on a 37 °C metal plate or in a 37 °C water bath for 5 min, and finally placed back on ice for 5 min. After the series of incubation treatments, 800 μL of antibiotic-free LB liquid medium was added to this mixture and placed in a shaker at 29 °C and 180 rpm for 2–3 h. About 200 μL of the obtained bacterial culture was spread on an LB solid medium containing kanamycin (50 mg/mL) and rifampicin (25 mg/mL) and incubated in a shaker at 29 °C for two days. The monoclonal colonies found growing on the solid medium were selected for PCR confirmation of the transformants. The confirmed positive colonies were finally inoculated in fresh LB liquid medium containing kanamycin and cultured to obtain the bacterial suspension for plant transformation.

### 4.3. Generation of Arabidopsis Thaliana Overexpressing PoWOX1

The Columbia wild-type (Col-0) Arabidopsis plants were transformed with the *Agrobacterium tumefaciens* GV3101 strain carrying the 35S::PoWOX1-EGFP plant expression vector using the dip-infiltration method. Approximately 10 μL of the bacterial culture was aspirated using a sterile syringe, inoculated into 5 mL of LB liquid medium (kanamycin), and incubated at 29 °C and 200 rpm for 24 h. The overnight culture was subcultured into fresh medium (1% ratio) and incubated for another 10–12 h. The inoculum was then transferred to 100 mL of LB liquid medium and incubated at 29 °C and 200 rpm until an OD_600_ of 1.0 was obtained. The obtained culture was centrifuged at 20 °C and 4000 rpm for 15 min to collect the bacterial pellet, which was resuspended in the transformation buffer [MS, sucrose 50 g/L, 6-BA (1 mg/mL), Silwet-L77 400 μL/L, pH 5.8]. The process was repeated, and the bacterial cells were pooled to obtain a suspension with an OD_600_ of 1.0.

The Arabidopsis plants were watered at the onset of flowering, and the open flowers and siliques of these plants were removed on the subsequent day. Then, all inflorescences were immersed in the bacterial suspension for about 1 min. Immediately after the treatment, the above-ground plant part was wrapped in a plastic film, and the plant was incubated in the dark to enhance infection. After 24 h, the plastic film was removed, and the plant was maintained in an artificial climate chamber at 24 ± 1 °C under normal light conditions. After seven days, the transformation procedure was repeated. About 2–3 weeks after the repeated transformation, the pods were collected, dried, and cracked to harvest the seeds, which served as the T0 transgenic seeds.

The T0 seeds were sown on ½ MS medium containing 30 mg/L hygromycin (Hyg) (8 g/L agar, without sucrose, pH 5.8), and the seedlings that produced green leaves were transplanted to soil. After 40 days, DNA was extracted from the seedlings, and PCR was performed using primers for the hygromycin resistance gene (HPT) (Table 1) to identify the positive T1 seedlings. The PCR involved a pre-denaturation step at 94 °C, followed by 35 amplification cycles of denaturation at 94 °C for 30 s, annealing at 60 °C for 30 s, extension at 72 °C for 1 min, and a final extension at 72 °C for 5 min. A total of 16 lines were identified as positive transformants at this stage. The T1 seeds were sown on ½ MS medium, and PCR was performed as mentioned above to identify the positive T2 seedlings, using three biological replicates per line. Eventually, eight homozygous T2 lines were identified, and the seeds from these lines were sown in pots to examine the plant phenotype; seeds of the wild-type plants were sown for comparison. Three T3 transgenic lines with significant differences in phenotype compared to the wild-type plants were selected to analyze plant growth and development and protein expression. A Zeiss Axio Zoom V16 fluorescence microscope (Zeiss, Beijing, China) at a wavelength of 509 nm and with a GFP filter was used to examine the fluorescence expression sites in the transformants and capture the images.

### 4.4. Phenotypic Analysis of Roots and Leaves Differentiated from Arabidopsis Healing Tissue

The seeds obtained from the T3 Arabidopsis lines and the wild-type plants were surface-sterilized with 75% ethanol for 1 min, followed by rinsing with sterile water 3–5 times. The sterilized seeds were sown on MS solid medium (4.4 g/LMS + sucrose 30 g/L + agar 9 g/L; 240 mL) in culture flasks and kept in a refrigerator at 4 °C for the first 2–3 days (vernalization) and a growth chamber with a relative humidity of 60–70%, a temperature of 24 ± 1 °C, a light/dark cycle of 16 h/8 h, and a light intensity of 40 μmol·m^−2^·s^−1^ for the subsequent 15 days. Ten seedlings with consistent growth were selected from each group, and the growth and development of the roots and leaves were analyzed.

Approximately 2–3 cm long roots and 1 cm × 1 cm leaf segments were collected from the transgenic and wild-type Arabidopsis plants and placed separately on callus induction medium (CIM) (B5 + 0.5 mg/L 2,4-D + 0.1 mg/L KT + 30 g/L sucrose + 2–4 g/L agar) and incubated for one week in the dark to induce callus formation. These calluses were transferred to a shoot induction medium (SIM) (B5 + 0.15 mg/L IAA + 0.5 mg/L 2-iPA + 10 g/L sucrose + 2–4 g/L agar) and maintained under normal light conditions for differentiation. 

### 4.5. Histological Analysis of Arabidopsis Roots

Tissues were collected from a region 3–5 cm from the root tip of the wild-type and *PoWOX1*-overexpressing Arabidopsis plants and fixed in formalin–acetic acid (FA) fixative for 24 h at 4 °C. After fixation, these root tissues were washed with 1× PBS and dehydrated using the following solutions in a dehydration machine (Donatello, DIAPATH, Milan, Italy): 75% ethanol for 4 h, 85% ethanol for 2 h, 90% ethanol for 2 h, 95% ethanol for 1 h, absolute ethanol I and absolute ethanol II for 30 min each, alcohol–benzene mixture, xylene I and xylene II for 5–10 min each, and molten paraffin I, molten paraffin II, and molten paraffin III at 65 °C for 1 h each. The dehydrated tissues were embedded in paraffin using an embedding machine (JB-P5; Wuhan Junjie Electronics Co., Ltd., Wuhan, China), and 4 μm thick sections were obtained using a microtome (RM2016; Shanghai Leica Instrument Co., Ltd., Shanghai, China). After allowing them to float on water, the sections were placed on clean glass slides at room temperature, air-dried at 42 °C, and stained with hematoxylin and eosin for 6 min. The stained sections were rinsed in water, re-stained for 15 s, again rinsed in water, and mounted with neutral gum. The sections were finally observed and photographed using a Zeiss Axio Zoom microscope.

### 4.6. RNA Extraction and Real-Time PCR

The roots, stems, leaves, flowers, and fruits were collected from the T3 Arabidopsis plants, and total RNA was extracted using the Quick RNA Isolation Kit (Hua Yueyang, Beijing, China). The extracted RNA was reverse-transcribed into first-strand cDNA with a reverse transcription kit (TaKaRa, Kusatsu, Japan). The obtained cDNA was used to determine the gene expression levels via real-time PCR with the TB Green Premix Ex Taq II fluorescence quantitative kit (Tli RNaseH Plus, TaKaRa) on a QTOWER real-time fluorescence quantitative PCR instrument (Analytik, Jena, Germany). The reaction mixture (10 μL) contained TB Green Premix Ex Taq (5 μL), a cDNA template (1 μL), forward and reverse primers (0.4 μL each), and ddH_2_O. Three biological replicates were maintained per reaction, and the PCR program was set as follows: pre-denaturation at 95 °C for 90 s, denaturation at 95 °C for 5 s, and annealing at 60 °C for 30 s for a total of 40 cycles. The melting curve was generated by running a program from 60 °C to 95 °C, with a 1 °C increase every 15 s. Finally, the relative expression levels of *PoWOX1* were calculated following the 2^−ΔΔCT^ method [49], using actin as the reference gene and the gene expression level in the roots of the Arabidopsis transformants as the control. The primers used in this assay are listed in Table 1.

### 4.7. Sequence Alignment and Phylogenetic Tree Generation

The amino acid sequences of the WOX proteins from 13 species, including Arabidopsis thaliana, Oryza sativa, Juglans regia, Vitis vinifera, Populus trichocarpa, Amborella trichopoda, Theobroma cacao, Picea abies, Selaginella moellendorffii, Ceratopteris richardii, Ginkgo biloba, Physcomitrella patens, and Ostreococcus lucimarinus, were retrieved from publicly available databases, namely, Ensembl Plants, the Plant Transcription Factor Database (PlantTFDB), and NCBI. Then, multiple sequence alignment was performed using Clustal W [50], and phylogeny was analyzed using the neighbor-joining method with 1000 bootstrap repetitions in MEGA 11 software [51], with default settings. Additionally, the amino acid sequences of WOX1 proteins from Oryza sativa, Arabidopsis thaliana, Juglans regia, Vitis vinifera, Populus trichocarpa, and Paeonia ostii were compared (multiple sequence comparison) and visualized using the Clustal W plug-in in DNAMAN 9.0 software. The online tool WEBLOGO (http://weblogo.berkeley.edu/logo.cgi (accessed on 24 October 2023)) was utilized to generate the sequence logos, and the MEME website (MEME-Submission form at meme-suite.org, accessed on 21 April 2024)) with the motif site distribution set to 0 or 1 and the motif count set to 10 was used to determine the conserved motifs. Finally, the TBtools software (v.2.4.0.119028) with the Gene Structure View (Advanced) plugin was employed to analyze and visualize the evolutionary tree and the motifs [52].

## 5. Conclusions

The present study proved that peony *PoWOX1* plays crucial roles in root development, callus tissue induction, and root differentiation in *Arabidopsis thaliana*. These findings provide novel insights into the molecular mechanism underlying regeneration in the peony. Therefore, we propose using *PoWOX1* as a candidate gene to develop an efficient peony regeneration system. However, future research should focus on analyzing other factors and hormones associated with PoWOX1 in regulating somatic embryogenesis in the peony.

## Figures and Tables

**Table 1:** Primers used for PCR in this study.

Primers	Primer Sequence (5′-3′)	Purpose
HPT-F	GGTCGCGGAGGCTATGGATGC	PCR identification of positive seedlings
HPT-R	GCTTCTGCGGGCGATTTGTGT	
Q-PoWOX1-F	CGTTGGCGGCAATGAAGAAGAATC	Real-time PCR
Q-PoWOX1-R	GGCAATTAGGAGGACTCAAGTTGGTAT	
Q-AtActin-F	GGTATGGGTCAGAAAGATGCT	
Q-AtActin-R	CGTTGTAGAAAGTGTGATGCC	

## Data Availability

All data in this study are available in the manuscript.
